# Investigation of the removal mechanism of antibiotic ceftazidime by green algae and subsequent microbic impact assessment

**DOI:** 10.1038/s41598-017-04128-3

**Published:** 2017-06-23

**Authors:** Ying Yu, Yangyang Zhou, Zhiliang Wang, Oscar Lopez Torres, Ruixin Guo, Jianqiu Chen

**Affiliations:** 1 College of Engineering, China Pharmaceutical University, 210009 Nanjing, China; 2 Jiangsu Key Laboratory of Environmental Engineering, Jiangsu Academic of Environmental Science, 210036 Nanjing, China

## Abstract

The present study provides an integrated view of algal removal of the antibiotic ceftazidime and its basic parent structure 7-aminocephalosporanic acid (7-ACA), including contribution analysis, bacteriostatic and aquatic toxic assessment and metabolite verification. 92.70% and 96.07% of the two target compounds was removed after the algal treatment, respectively. The algal removal can be separated into three steps: a rapid adsorption, a slow cell wall-transmission and the final biodegradation. Additionally, while ceftazidime demonstrated an excellent inhibitory effect on *Escherichia coli*, there was no bacteriostasis introduced after the algal treatment, which could avoid favoring the harmful selective pressure. On the other hand, no significant aquatic impact of the two target compounds on rotifers was observed and it was not enhanced after the algal treatment. To better reveal the mechanism involved, metabolite analyses were performed. Δ-3 ceftazidime and trans-ceftazidime were regarded as the metabolites of ceftazidime and the metabolite of 7-ACA was regarded as a compound which shared the similar structure with 4-chlorocinnamic acid. Our study indicated that the green algae performed a satisfactory growth capacity and played a dominant role for the biodegradation of the target antibiotics, which achieved high removal efficiency and low environmental impact.

## Introduction

The production, widespread and uncontrolled usage of antibiotics contributes to the presence of these compounds in aquatic environments^1^. Since 1982, several widely used antibiotics including macrolides, tetracyclines and sulphonamides have been confirmed in the environment^1^. Recently, more residues from human and/or veterinary antibiotics have been widely detected in animal tissues, treated sewage, industrial effluent, surface water bodies and hospital effluents^1, 2^. Antibiotic pollution in various environmental media is a serious and urgent issue due to their immediate and potential threats to environmental safety and human health^3–6^. Environmental exposure to antibiotics may accelerate the persistence or emergence of antibiotic resistance genes (ARGs)^7–10^, which could eventually lead to the ineffective treatment of disease^11^.

Strategies to control this contamination have received increased attention in recent years. Chemical oxidation processes usually achieve lower mineralization of target compounds and the reaction products of the treatment are usually more toxic than their parent compounds^12, 13^. On the other hand, biological treatments, such as activated sludge processes, are a widely and universally used technology in current sewage treatment plants (STPs)^14^. Two main processes, adsorption and bio-degradation by active sludge, are responsible for the fates of organic contaminants in sewage. Due to the impact of antibiotics on microbes, their biodegradation efficiency was limited when the initial concentrations increased^1^. Desorption was also observed reversibly from sludge after adsorption^15^. The above biological treatment, regardless of its efficiency, leads to the production of antibiotic-resistant bacteria in the final effluents, sometimes at higher percentages that in raw inflow^16, 17^. Thus, a greener and safer solution must be introduced to control the problem of antibiotics in the environment.

Compared with the activated sludge, microalgae have the greatest abundance of plant biomass in aquatic environments and a higher tolerance to contaminants than bacteria^18^. Microalgae play an important role in maturation ponds, and in domestic, facultative or aerobic ponds, that treat small- and middle-scale municipal wastewater, due to their excellent ability to remove nutrients, heavy metals and pathogens^19^. As the non-target organism, green algae have higher tolerance to antibiotics than other algal species and high removal efficiency for antibiotics^20–23^.

Although there were many good applications of the green algae to treat antibiotic, most previous studies focused on the removal efficiency of the target compounds, while the characteristics of the algae during the treatment were less characterized. Secondly, an excellent removal capacity is not valuable enough to justify algal treatment. A green sustainable biotechnology should provide not only high removal efficiency, but also low overall environmental impact. Because the conventional biological processes are suspected to contribute to antibiotic-resistant bacteria selection and resistance transfer among bacteria^24^, whether the green algae exerted a similar selective pressure should be determined. Thus, a bacteriostatic assessment of the target antibiotic was performed before and after the algal treatment. On the other hand, previous studies also indicated the impact of antibiotics on the non-target organisms^25, 26^. Thus, to exclude the possibility that the toxicity of the target antibiotics increased after the algal treatment (as in UV irradiation or other chemical degradation^12^), the aquatic impact of the target antibiotics and the corresponding effluent after the algal treatment was also assessed using rotifers as the test organism^27^.

**Figure 1:**
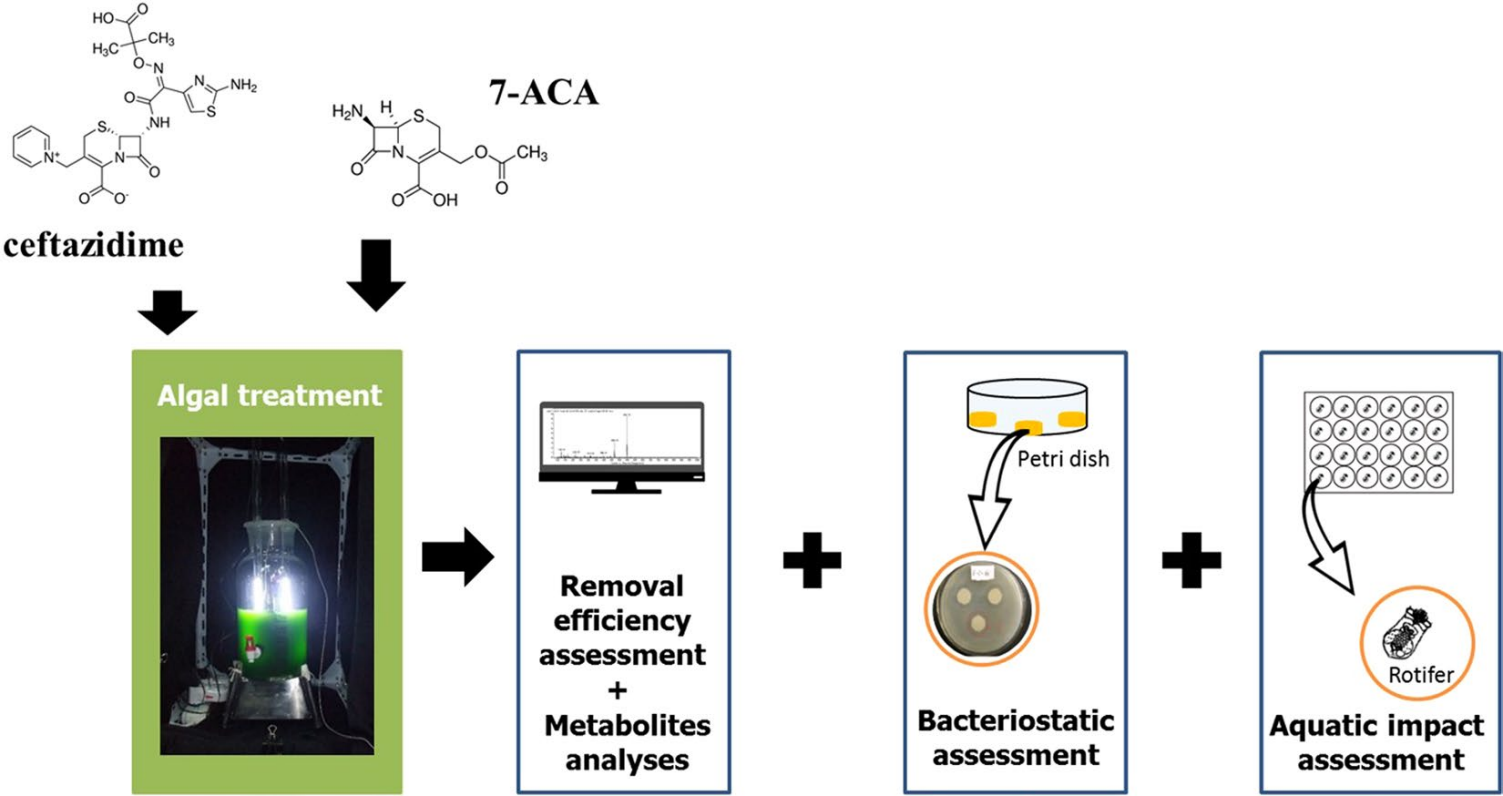
Schematic view of algal treatment and assessment processes.

Ceftazidime, one of the most widely used cephalosporins in sewage, surface waters and drinking waters^28^, was selected as the target antibiotic in the present study. 7-aminocephalosporanic acid (7-ACA), the basic parent structure of ceftazidime, occurs widely in the manufacture of this antibiotic and its production wastewater. Thus, 7-ACA was also used as another target antibiotic. The aim of the present study was to provide an integrated view of a algal removal of the two target antibiotics, ceftazidime and 7-ACA. The first mission of our study was to demonstrate the characteristics of the algal treatment on the antibiotics, including the algal growth capacity, removal efficiency and the treatment step classification. Secondly, the bacteriostatic assessment and rotifer toxicity of the target compounds before and after the algal treatment was set-up to assess this environmental impact. Additionally, to better understand the mechanism involved in, the structure of the metabolites after the action of the green algae on the target compounds was analyzed. Thus, compared with the previous research, our study provided an integrated view which could indicate that the algal removal of the antibiotics is a green sustainable biotechnology with high removal efficiency and low environmental impact (See in Fig. 1).

## Results

### Algal growth capacity and removal efficiency assessment

**Figure 2:**
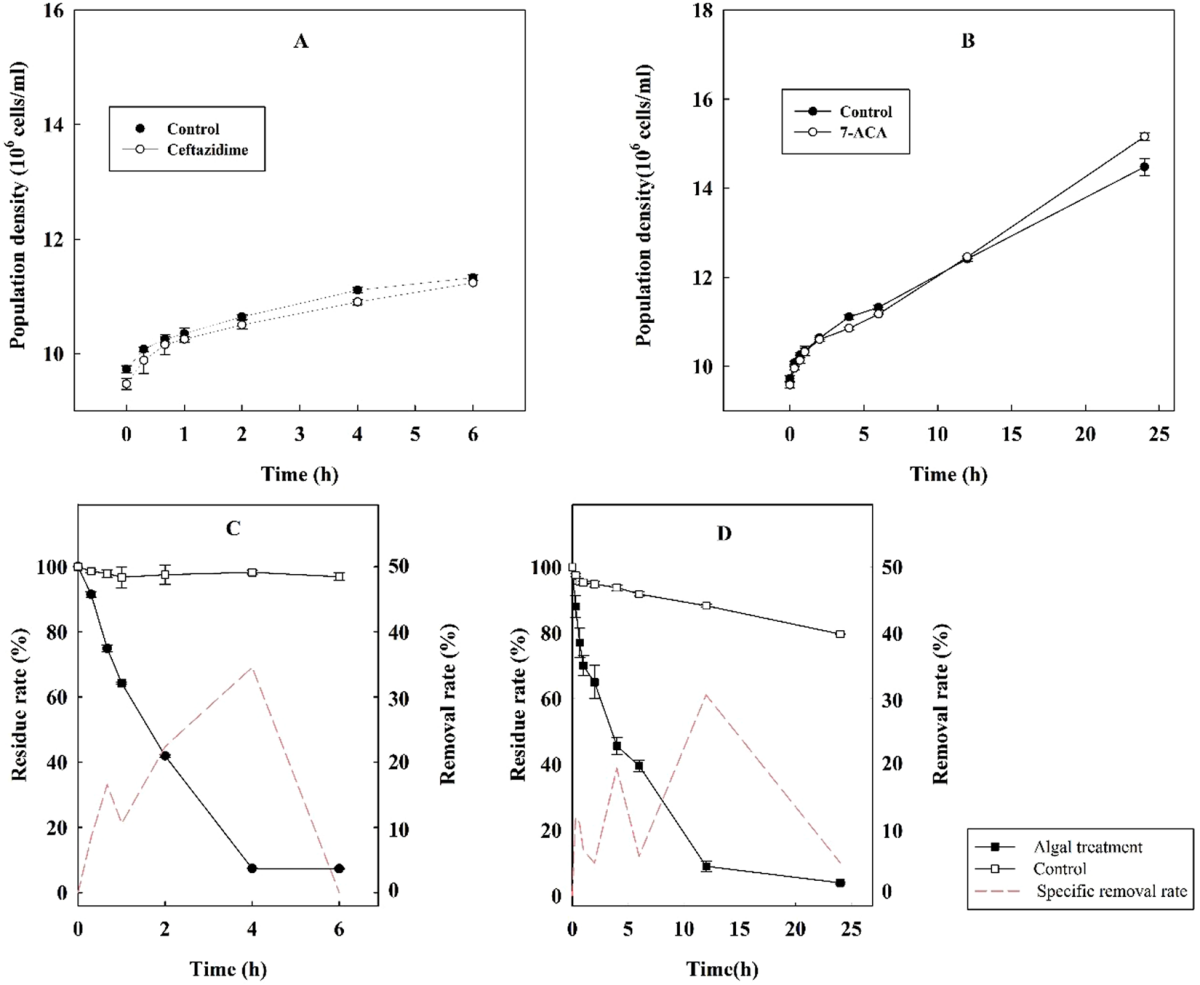
Growth capacity and removal efficiency of green algae on ceftazidime (**A**) and 7-ACA (**B**). Residue rate of ceftazidime (**C**) and 7-ACA (**D**) during algal treatment.

Ceftazidime typically inhibits the synthesis of peptidoglycan in the bacterial cell wall, while green alga is not the target organism of the compound^29^. The aim of the present study was to provide an integrated view of algal removal of an antibiotic. The algal growth capacity in the presence of the given compound is therefore the premise of the algal treatment. Population growth curves for the green algae under ceftazidime and 7-ACA are presented in Fig. 2A and B. Generally, the algae exhibited satisfactory growth capacity during the entire treatment process (6 h for ceftazidime and 24 h for 7-ACA), regardless of the compound. The algal population density at the time points was 9.47 ± 0.10, 9.88 ± 0.23, 10.16 ± 0.18, 10.25 ± 0.05, 10.90 ± 0.05, 11.24, 12.43 ± 0.05 and 14.96 ± 0.03 × 10^6^ cells/mL, which was 97.38%, 98.08%, 99.06%, 99.06%, 98.69%, 98.14% and 99.20% of the control. This indicated that *C*. *pyrenoidosa* was tolerant and able to grow in the presence of ceftazidime. The same conclusion was obtained when green algae was used to treat 7-ACA. The algal population density was 12.45 and 15.16 × 10^6^ cells/mL, which was 1.46% and 5.86% more than the control.

The results in Fig. 2C indicated that the proportion of self-degradation and the algal removal of the target compounds were significantly different during algal treatment. The residue rates for the target antibiotics and the contributions of the algal removal all changed with the treatment period. For ceftazidime, 91.53% and 7.35% of the antibiotic was detected at 20 min and 6 h, respectively, revealing that the total removal rate increased rapidly from 8.47% to 92.70% during the treatment. The concentration changed little under self-gradation (Fig. 2C, control); 96.93% of ceftazidime was detected after 6 h, which indicated that the compound was resistant to degradation if algal action was absent. For 7-ACA, our preliminary experiment showed that approximately 40% of the compound was still detected after 6 h. The algal treatment of 7-ACA was therefore extended to 24 h to obtain a better removal efficiency and more analyzable metabolite. Although 20% of 7-ACA could be removed by self-degradation, the rapidly decreased residue rate of the compound in the algal treatment system indicated that the green algae also had a good removal capacity for 7-ACA. As shown in Fig. 2D, only 3.93% of the compound remained at 24 h.

### Bacteriostatic and aquatic impact assessment

**Figure 3:**
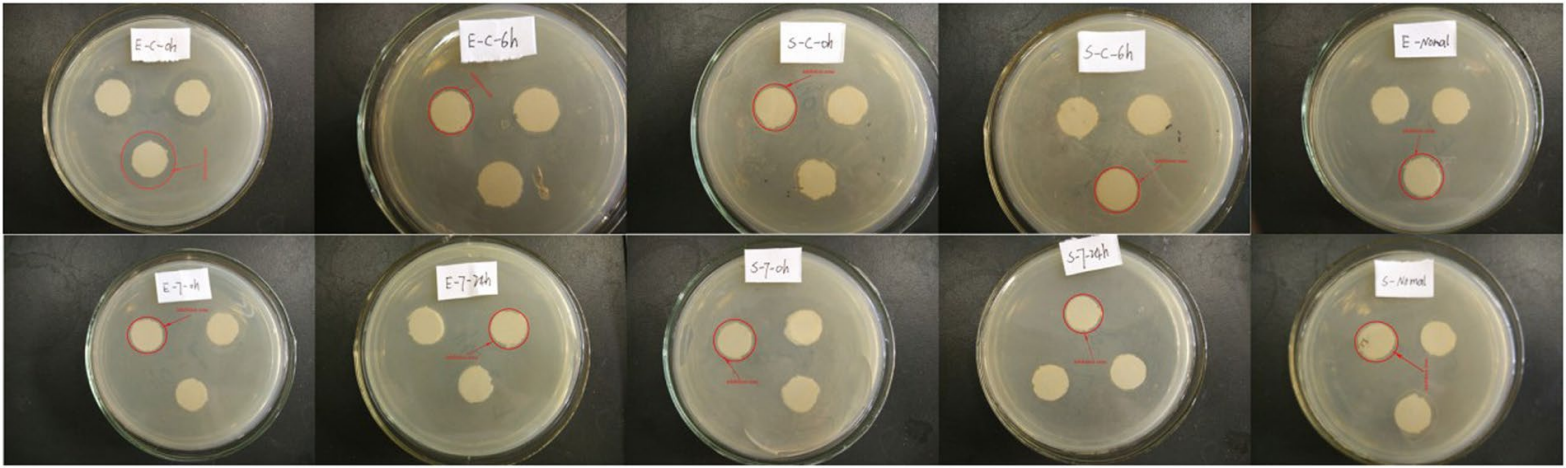
Inhibition zone of two bacterial species *E*. *coli* and *S*. *aureus* under target compound (ceftazidime and 7-ACA) and corresponding algal metabolites, respectively. The two bacteria species cultured with algal media without any compound was used as a control.

Due to the antibiotic residues and/or other substances, STPs are considered the major driving force in the antibiotic resistance selection and spreading^30, 31^, regardless of efficiency or operational conditions. Green algae are not the target organisms of antibiotics. In the present study, the green algae *C*. *pyrenoidosa* had good tolerance and a high removal capacity during the treatment period. However, whether algal treatment applies a selective pressure should be studied, regardless of the high removal efficiency. Thus, in our study, we performed a bacteriostatic assessment of the target compounds (ceftazidime and 7-ACA) before and after the algal treatment. Ten groups (see in Table 1) were designed to detect the effects of ceftazidime or 7-ACA, and the metabolites after algal treatment were studies in two bacteria species *E*. *coli* (gram-negative bacteria) and *S*. *aureus* (gram-positive bacteria). The inhibition zones of the two bacteria species in the presence of these compounds are presented in Fig. 3. The width of the inhibition zone generally indicates the bacteriostatic level of the testing sample. In the present study, the widest inhibition zone was observed for the group marked E-C-0, which determined the bacteriostatic activity on *E*. *coli*. Our result was supported by a previous publication that reported that ceftazidime exerted excellent bacteriostasis on gram-negative bacteria^32^. In the present study, the two bacteria species cultured with the culture solution of the alga without either antibiotic were used as a control. Small equally sized inhibition zones were obtained in most groups. Our results reflected a number of conclusions as follows: (1) the bacteriostasis of 7-ACA was not noticeable in either *E*. *coli* or *S*. *aureus* and the antibacterial activity was also not improved by the algal treatment. (2) After a 6-h algal treatment, the level of ceftazidime had decreased to below the minimum inhibitory concentration (MIC), while the corresponding algal metabolite lacked antibacterial activity.

**Table 1:** Mark of the bacteriostatic assessment.

Group mark	Bacteria species	Target compound	Treatment time
E-C-0h	*E*. *coli*	ceftazidime	0 h
E-C-6h	*E*. *coli*	ceftazidime	6 h
S-C-0h	*S*. *aureus*	ceftazidime	0 h
S-C-6h	*S*. *aureus*	ceftazidime	6 h
E-7-0h	*E*. *coli*	7-ACA	0 h
E-7-24h	*E*. *coli*	7-ACA	24 h
S-7-0h	*S*. *aureus*	7-ACA	0 h
S-7-24h	*S*. *aureus*	7-ACA	24 h
S-normal	*S*. *aureus*	—	—
E-normal	*E*. *coli*	—	—

**Figure 4:**
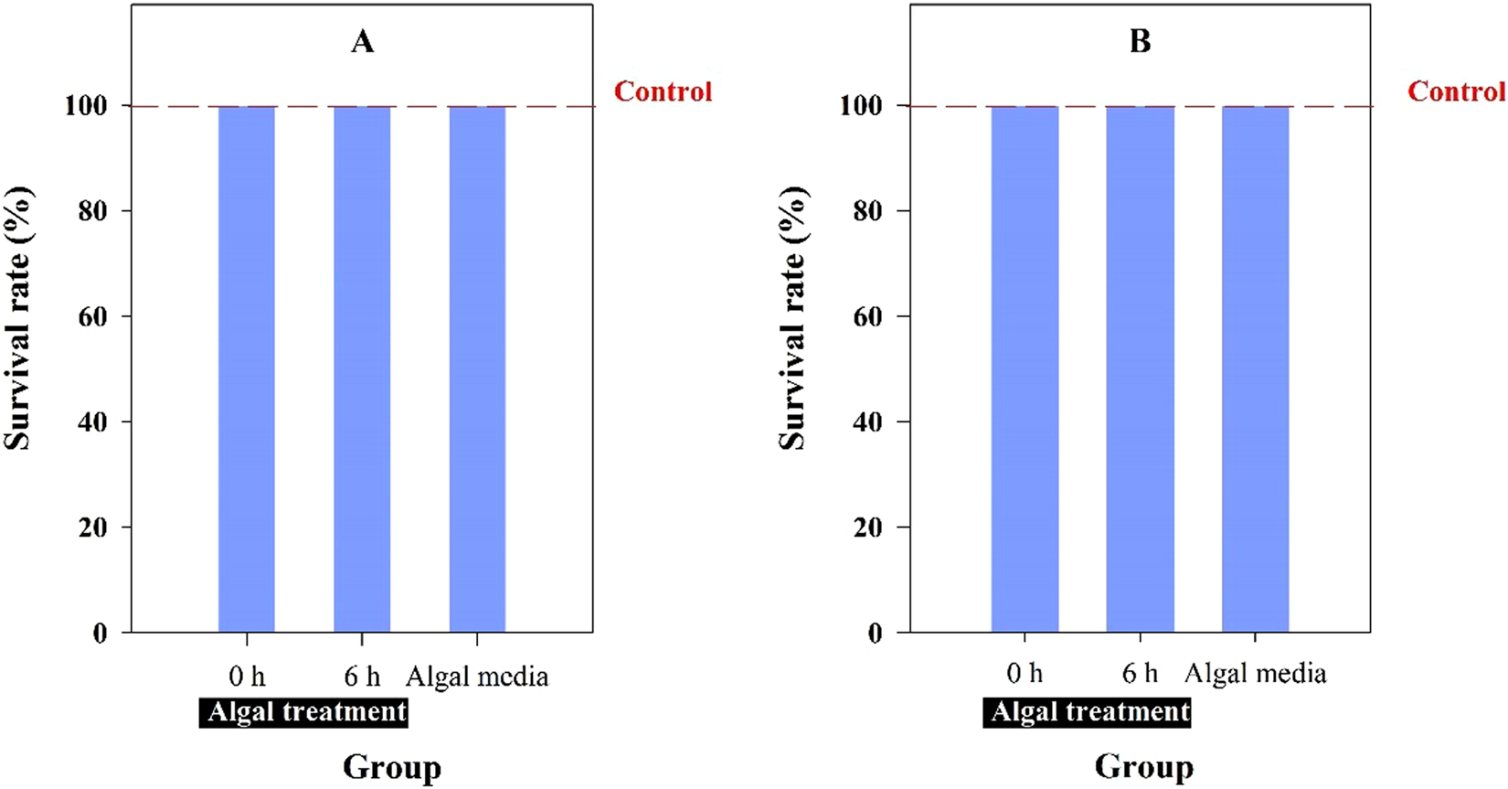
Acute (24 h) toxicity tests with rotifer *B*. *calyciflorus* under ceftazidime (**A**) and 7-ACA (**B**) and corresponding metabolites after algal treatment. The rotifer was cultured under EPA media as a control.

Advanced oxidation processes (AOPs) have proven to be highly effective in the degradation of most wastewater pollutants^33^. Unfortunately, apart from the disadvantage of the high operating costs and secondary pollution arising from the residual chemical, the partial oxidation of organic contaminants may result in the formation of intermediates that are more toxic than the parent compounds^12^. Therefore, the toxicity assessment of reaction products after algal treatment is of great importance. Invertebrates are commonly used in the evaluation of the toxic effects of pollutants in aqueous matrices. Aquatic toxicity assessment has also been considered and implemented. Acute (24 h) toxicity tests with rotifers are presented in Fig. 4. All test organisms survived when exposed to ceftazidime, 7-ACA and the corresponding algal metabolites. This implied that there was no significant toxicity of ceftazidime or 7-ACA on the rotifers, and the impact was not enhanced by algal treatment.

### Metabolites analyses

**Figure 5:**
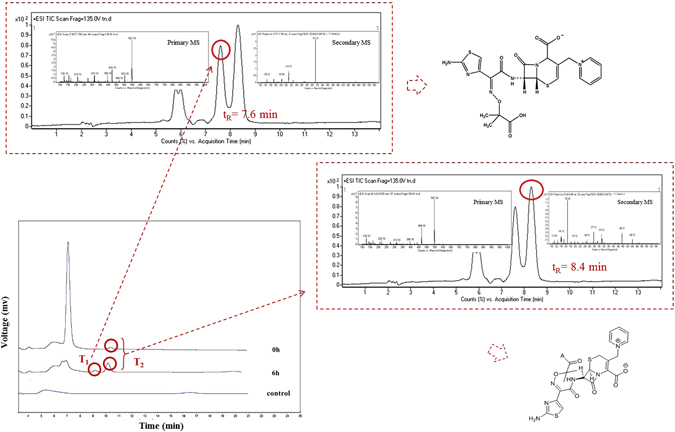
Chromatograms of ceftazidime before and after algal treatment (0 and 6 h, respectively). T_1_ and T_2_ are marked for the two metabolites after the action of green algae. A sample of algal media without the antibiotic was analysed as a control.

High removal efficiency with low environmental impact after treatment process is the critical characteristic in the consideration of algal removal treatments as a green sustainable biotechnology. To better reveal the mechanism involved, metabolite analyses were performed as the target compound underwent the algal treatment. The chromatograms of the antibiotic, sampled before and after algal treatment (0 and 6 h, respectively) are presented in Fig. 5. A new chromatographic peak was detected at a retention time of 9.2 min when ceftazidime underwent a 6-h algal treatment. Another chromatographic peak was also detected at a retention time of 10.2 min before algal treatment (0 h), while the peak area markedly increased at the same retention time after treatment. Because green algae might secrete active compound(s) in the treatment system after 6 h, even without antibiotics, the algae culture solution in the treatment system without antibiotic was analysed as the control. Compared with the control, the concentration of ceftazidime decreased, and two metabolites (T_1_ and T_2_ for short) were produced by the action of the green algae. For further analysis, LC-MS was employed to obtain structural information for the metabolite. The primary mass spectrum, product ion and structural formula graphs for the metabolite T_1_ and T_2_ of ceftazidime after algal treatment are presented in Fig. 5. According to the mass spectra, the corresponding ion was observed at *m/z* 547, approximating the relative molecular mass of ceftazidime (546). Thus, T_1_ and T_2_ were viewed as isomers of ceftazidime. Additionally, the primary mass spectrum showed that the ions of T_1_ [M + H]^+^, [M + 2H]^2+^ and [M + 3H]^3+^ were observed at m/z 547.1, 548.1 and 549.1, respectively, and the most abundant fragment ions were observed at m/z 424.1, 313.1, 210.1 and 126.1, respectively. In previous studies^34, 35^, these structural changes were similar to the decomposition behaviour of Δ-3 ceftazidime, a Δ-3 isomer of ceftazidime. The compound is the impurity produced from ceftazidime when the double bond of carbon is translocated from the 2-position to the 3-position. Based on previous research, the characteristic fragment ions of Δ-3 ceftazidime were *m/z* of 468 and 313 (data from a doctoral thesis from Peking University Medical College, unpublished), the metabolite T_1_ (t_R_ = 7.6 min) in the present study is therefore regarded as Δ-3 ceftazidime. The molecular ions of T_2_ were observed at *m/z* 547.1, 548.1 and 549.1, and the product ions were observed at *m/z* 468.1, 396.1, 313.1, 293, 277.1, 167 and 139. Ceftazidime and trans-ceftazidime are cis-trans isomers that undergo different fragmentation pathways. The characteristic fragment ions of ceftazidime were of *m/z* 293, 292 and 277, but that for trans-ceftazidime were 293, 292 and 265. The product ions with *m/z* values of 293 and 292 in trans-ceftazidime had a higher abundance than for ceftazidime. Because the retention time of ceftazidime was 6.1 min (not present in the Fig. 5), T_2_ (t_R_ = 8.4 min) described above is regarded as trans-ceftazidime.

**Figure 6:**
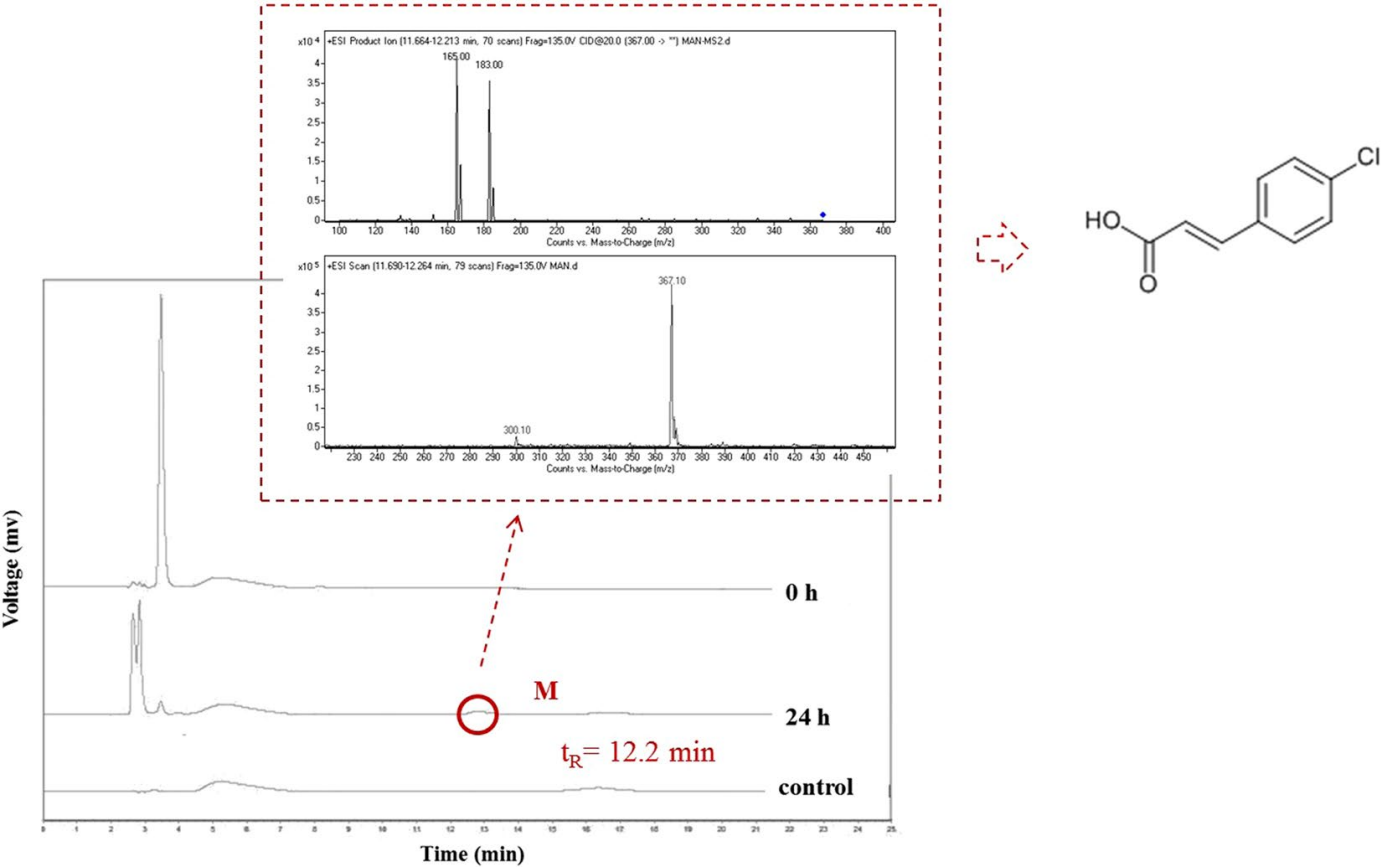
Chromatograms of 7-ACA before and after algal treatment (0 and 12 h, respectively). M was marked for the metabolite after the action of green algae. A sample of algal media without the compound was analysed as a control.

As the basic parent structure of ceftazidime, 7-ACA is usually detected in the wastewater of ceftazidime production. Thus, the removal mechanism of 7-ACA by green algae was also considered and evaluated. The chromatograms of the antibiotic, sampled before and after algal treatment (0 and 24 h, respectively), are presented in Fig. 6. Among the three groups, a new chromatographic peak was detected at a retention time of 12.2 min, which indicated that a new metabolite was produced (mark M for short) after the action of green algae. According to mass spectra, the ions of M [M + H]^+^, [M + 2H]^2+^ and [M + 3H]^3+^ were observed at *m/z* 367.1, 368.1 and 369.1, respectively, and the most abundant fragment ions were observed at *m/z* 185, 183, 167 and 165. The relative abundance ratio of *m/z* 185 to *m/z* 183 was 1:4.3, while that of *m/z* 167 to *m/z* 165 was 1:2.9. Based on the results, the relative molecular weight of M was 366 and a helium atom was involved in the structure of the compound. According to information provided from the Chemistry Database (http://sdbs.db.aist.go.jp/sdbs/cgi-bin/direct_frame_top.cgi), The M described above might be regarded as a compound similar in structure to 4-chlorocinnamic acid (C_9_H_7_O_2_Cl). Biodegradation is usually more complex than chemical degradation. Based on our current technology and data, we considered possible structures. More accurate structures and potential degradation (conjugation) mechanisms should be considered in the future. More advanced analytical methods and algal metabonomics techniques should be applied to better deduce the biodegradation mechanism.

## Discussion

Although the green algae is not the target organism of the antibiotic, several previous studies demonstrated the impact of antibiotics on aquatic species, especially on the algae^29, 36, 37^. The algal population growth could be inhibited when the photosynthesis was disturbed^37, 38^. Thus, the impact of the compounds on algae is one of the limiting factors in the algal treatment application and whether algae maintain the growth capacity during the treatment is therefore the premise of the algal removal. Our results indicated that the population of the green alga tended to increase until the end of the treatment, regardless of the compound (Fig. 2A and B). The maximum of the algal density under the two target compounds was 99.20% and 105.86% of the control alga at the end of the treatment, respectively. Our results indicated that the green alga had a satisfactory growth capacity during the whole treatment process. It’s worth noting that the algal population density in the treatment was more than that of the control. The results indicated that 7-ACA (40 mg/L) could improve the growth of algae during the treatment. Previous study indicated that three lipid-rich microalgae examined exhibited relatively high resistance to 7-ACA at 100 mg/L, and a slight inhibition effects on the microalgal growth (9.6–12.0%) was observed^39^. The effect of 7-ACA on the algae was characterized as the change from a low-dose stimulation to a high-dose inhibition. It might be viewed as a hormetic effect or the difference in species-dependent response.

Antibiotic structure is usually changed via abiotic processes or biotic inducement. Previous research has shown the elimination of target compounds by multiple abiotic processes such as sorption, photodegradation and hydrolysis^40^. Hydrolysis and photolysis usually serve as the predominant pathways of abiotic degradation^41^. Thus, it should be considered whether self-degradation of ceftazidime and 7-ACA occurred and led to the actual removal of target compounds. The residue rates in the presence of the algal treatment or absence (Fig. 2C and D), indicated that 7.35% of ceftazidime and 3.93% of 7-ACA remained after a 6-h algal treatment, while the corresponding results were 96.93% and 79.66%, respectively, when the alga was absent. The results implied that most of the total removal efficiency is attributed to the green algae. Thus, green alga plays a dominant role in the removal of target compounds in the present treatment system. Previous researches supported a similar conclusion. The chlorophycean *C*. *vulgaris* demonstrated an efficient uptake capacity of tetracycline^21^ and norfloxacin^22^. And 32.9% of spiramycin and 33.6% of amoxicillin could be eliminated in the presence of *M*. *aeruginosa* ^23^.

As an important component of primary production in aquatic environments, algae usually have excellent removal capacities for the contaminants. Although a high removal efficiency was obtained in our algal treatment system, we focused on the mechanism involved. Among inorganic pollutants, heavy metals are non-biodegradable and can accumulate in organisms. Based on their large surface-to-volume ratio, algae have been investigated as a biosorbent for the removal of heavy metals^42^. Functional groups, such as amino, carboxyl, hydroxyl and carbonyl groups, on the surface of this biomass are responsible for biosorption. Removal of synthetic organic compounds by microalgae has also been widely demonstrated, including fluoranthene^43^, tributyltin^44^, phenanthrene^45^ and other several polycyclic aromatic hydrocarbons (PAHs)^46^, especially antibiotics such as tetracycline^21^, norfloxacin^22^ and spiramycin^23^. Although the high surface area to volume ratio (S/V ratio) of the algae usually possesses a high potential for sorption, a previous study indicated that the relationship between the percentage of PAH accumulation and the S/V ratio was not significant^46^. Additionally, algal cell walls possess an overall negative charge and have receptors capable of binding and attracting cations^47^. Due to their strong binding affinity, microalgae cell walls, consisting of cellulose and lipid, also affect the biosorption processes^48^. For this reason, previous research also evaluated the removal efficiency of the target compound in the live and dead microalgae cells. The percent of TBT removed by live cells was significantly lower than in dead cells of the same algal species in a three -days treatment^44^. The enhanced organic compound sorption of dead biomass was explained by the loss of permeability control when the cell membrane structures was destroyed^49^ or by additional membrane-bound organelles and other intracellular compounds that are exposed following cell lysis^50^. To better understand the mechanism involved in the present algal treatment process, the removal rate for the target antibiotic by dead and living algal cells was compared with our preliminary experiment. Our results indicated that the living algal cell had a better removal capacity, and in fact, was double that of a dead cell. This suggests that the mechanism in the removal of the target compound by microalgae was more complex for heavy metals. The previous publication demonstrated uptake of TBT by the algae *C*. *emersonii* in two phases: a rapid initial uptake phase within 5 min and a slower and apparently linear uptake after 2 h^51^. In the present study, the specific removal rate at each time point of the algal treatment was considered and evaluated. The slope of the red short dashed line (in Fig. 2C and D) indicates the specific removal rate in a given period. For ceftazidime, a high removal rate was obtained in the first 40 min, followed by a decreased rate after 1 h. The previous study suggested that cell walls act as a frontier barrier for access of TBT to the cell interior. Once inside the cell, TBT is a membrane-active compound and is transported into intracellular fractions via ion channels or carriers in the cell membrane. Another study showed that polychlorinated biphenyl (PCB) uptake in phytoplankton occurred through cell-membrane diffusion^52^. Based on the above results and conclusions, the removal process of green algae on ceftazidime could involve a rapid initial removal via passive physicochemical adsorption (the first peak at 40 min) and a slow cell wall-transmission process (after 1 h). The disappearance of TBT after adsorption suggests biodegradation; photoautotrophic marine algae are also known to metabolize naphthalene into a series of metabolites^53, 54^. Although several applications of algae exist to treat antibiotics, the algal degradation products from the corresponding target compound have been less characterized, which limits mechanistic studies. Biosorption refers to the physical-chemical adsorption that occurs at the cell surface and is metabolism-independent. The process therefore could occur in both live and dead cells. Thus, we suggest that the additional removal capacity of living algae above dead algae should be attributed to biodegradation.

Activated sludge is widely used in the biological treatment process^55^. For antibiotics, a compound that strongly impacts microorganisms usually leads to low treatment efficiency. Previous researches showed that treatment, regardless of efficiency or operational conditions, leads to the production of final effluents containing antibiotic-resistant bacteria. Although the target compounds ceftazidime and 7-ACA could be degraded during algal treatment, the environmental safety risk assessment of the effluent should also be considered. Our bacteriostatic assessment indicated that unlike the traditional biological treatment process, the conditions offered to microorganisms after an algal treatment process may not favor the selective pressure. In some cases, antibiotic-resistant bacteria were present at higher percentages in the effluent discharge than in the raw inflow^17, 56^. In contrast, green algae exert an advantage including high removal efficiency for the target compound and no bacteriostasis introduced for the final effluent. On the other hand, our previous report revealed the algal contribution in a combined UV-algae treatment to remove the antibiotic cefradine. The algal treatment step reduced the toxicity of the antibiotic from 93% to 55% (based on the death rate of rotifer)^27^. Although our present results could not demonstrate the direct detoxification on ceftazidime by green algae, it indicated that algal degradation of the parent compound avoided further by-product toxicity compared to chemical treatment.

Additionally, due to the high tension of *β*-lactams involved in the structure of cephalosporin, the compounds are easily influenced by external factors, and isomerization subsequently occurs. The Δ-3 isomer is therefore the product when the carbon-carbon double bond of cephalosporin is translocated. The antimicrobial activity of cephalosporin is related to the basic parent structure, particularly the carbon configuration at the given position and the location of the carbon-carbon double bond. Thus, compared with cephalosporin, the corresponding Δ-3 isomers have no obvious effect on pharmacodynamics evaluation parameters and are usually controlled as an impurity in the manufacturing process^35^. Notably, the metabolite of 7-ACA was regarded as a chlorinated compound with a similar structure to C_9_H_7_O_2_Cl. Other previous research has noted that halogenated compounds are produced by marine algae^57^. Biodegradation is affected by molecular weight, water solubility and lipophilicity of the target compound. Existing literature reports that poly-aromatic hydrocarbons, which have more rings, are more easily removed than monoaromatic compounds^58^ and poly-aromatic hydrocarbons with high molecular weight are preferentially degraded^43^. When we compared the specific removal rate of the two target compounds during the same period (0–6 h, red short dashed line in Fig. 2C and D), ceftazidime was faster and more easily removed by green algae than 7-ACA. This may have occurred because ceftazidime contains more rings and has a higher molecular weight than 7-ACA. Thus, our results imply that the second treatment step after adsorption is a slow, metabolism-dependent active uptake process that is strictly related to living organisms.

Although several applications of the green algae to treat antibiotics have been republished. The questions such as the long hydraulic retention time (HRT)^59^ and temperature-dependent removal efficiency should also be considered and solved^60^. Our study indicated that the algal removal of antibiotics could be viewed as a green sustainable biotechnology with high removal efficiency and low environmental impact. How to design an appropriate algal reactor in larger scale and how to regulate and control the condition of the algal reactor to obtain the high removal efficiency in a relative shorter HRT should be considered in our follow-up study for the application.

In conclusion, the present results not only reveal the high removal efficiency of green algae for the target antibiotic ceftazidime and its basic parent structure, 7-ACA, but also contribute to bacteriostatic, aquatic toxic assessment methods and preliminary analysis of the metabolites. Most of the two target compounds, ceftazidime (92.70%) and 7-ACA (96.07%) was removed after a 24 and 6 h, respectively. Compared with the previous studies, the present study demonstrated the detailed characteristics of an algal treatment on the target compounds. Three different steps of the algal treatment process have been confirmed. Understanding the metabolic process involved in algal treatments will assist in resolving the serious treatment problems of traditional bio-treatment technology. Based on the biodegradation, our study demonstrates that using green algae to treat antibiotic is promising for the application due to the potential of high removal efficiency and low environmental impact.

## Materials and Methods

### Test materials and culture conditions

The algae *C*. *pyrenoidosa* was obtained from the Institute of Hydrobiology of the Chinese Academy of Sciences (China), and cultivated in Blue-Green medium (BG-11): 1.5 g/L of NaNO_3_; 0.04 g/L of K_2_HPO_4_; 0.075 g/L of MgSO_4_·7H_2_O; 0.036 g/L of CaCl_2_·2H_2_O; 0.006 g/L of citric acid; 0.006 g/L of ferric ammonium citrate; 0.001 g/L of EDTANa_2_; 0.02 g/L of Na_2_CO_3_; 1 mL/L of trace metal solution (A_5_, composed of 2.86 g/L of H_3_BO_3_, 1.86 g/L of MnCl_2_·4H_2_O, 0.22 g/L of ZnSO_4_·7H_2_O, 0.39 g/L of Na_2_MoO_4_·2H_2_O, 0.08 g/L of CuSO_4_·5H_2_O and 0.05 g/L of Co(NO_3_)_2_·6H_2_O). The cultural condition was maintained at 25 ± 1 °C in an illumination incubator with a 12:12 h light/dark cycle.

The *E*. *coli* and *S*. *aureus* used in the experiment were donated from The College of Life Science and Technology, China Pharmaceutical University. The medium for cultivating *E*. *coli* and *S*. *aureus* was beef extract peptone medium (3 g of beef extract; 10 g of peptone; 5 g of NaCl; 16 g of agar; 1000 mL of water; pH: 7.4–7.6). The bacteria were cultivated at 37 °C in a bacteriological incubator. The freshwater rotifer *B*. *calyciflorus* was obtained from a pond on the Jiangning campus of China Pharmaceutical University. The population was cloned from a single female and maintained in our laboratory for three months. The rotifer was cultured in EPA medium (96 mg of NaHCO_3_, 60 mg of MgSO_4_, 60 mg of CaSO_4_, 4 mg of KCl, 1000 mL distilled water, pH: 7.5) and the green alga *C*. *pyrenoidosa* was used as the food. The rotifers were maintained at 25 ± l °C on a 12:12 h light/dark interval in the illumination incubator (GZP-300B) with 2000 lux light. The medium and food were renewed daily.

### Chemical and analytical methods

The antibiotic ceftazidime (>98% purity) and 7-ACA (>99 purity) used in the experiment were purchased from Yabang Investment Holding Group Co., LTD, China (more details in Table 2). The drug concentrations were determined using high-performance liquid chromatography (HPLC, LC-10AT, Shimadzu). Ceftazidime and 7-ACA were separated and determined using an Inertsil ODS column (4.6 mm × 150 mm, 5 *μ*m). The mobile phase of the two antibiotics was as follows: methanol-0.1% formic acid (20:80) for ceftazidime and methanol-0.1% formic acid (25:75) for 7-ACA. The flow rates were 1.5 mL/min and 0.5 mL/min, respectively, under ambient temperature. All detections were performed using 254 nm UV absorption. The retention times of ceftazidime and 7-ACA were 3.4 min and 3.9 min, respectively. Quantization was performed using external standards and was based on peak areas. The limit of detection and limit of quantitation (0.0193 mg/L and 0.0585 mg/L, respectively) of the analytical methodology were three times and ten times the standard deviations of the background noise, respectively.

**Table 2:** Basic information of the selected target compounds in the algal treatment.

Name	Molecular Formula	Molecular Weight	p*K*_a_	Structural Formula
Ceftazidime	C_22_H_22_N_6_O_7_S_2_·5H_2_O	636.7	1.9	
			2.7	
			4.1	
7-ACA	C_10_H_12_N_2_O_5_S	272.27	4.8–4.9	

### Experimental set-up

Before the experiment, algae were cultivated to the exponential growth phase in BG-11 media. The main experiment was divided into two parts. In Part I, algae in the exponential growth phase was used to treat ceftazidime and 7-ACA, respectively. The process was performed in a 1-L photoreactor (Fig. 1) and conducted at 25 ± 1 °C under continuous illumination. The initial algal density and the concentration of the two target compounds in the treatment were 10 × 10^6^ cells/mL and 40 mg/L, respectively. The concentration of residual antibiotics in the treatment system was determined by HPLC at several checkpoints (0, 20 and 40 min, 1, 2, 4 and 6 h) during the treatment process and the results were used to calculate the residue rate. The specific removal rate was calculated as the compound removal rate within the unit interval. The samples were purified with disposable with disposable filters. The algal density was counted at the corresponding time to evaluate the growth capacity of the green algae. The concentrations of ceftazidime and 7-ACA without algae were also measured to evaluate any antibiotic self-degradation. The samples were withdrawn from the photoreactor with SPE (Supelco-12, USA) at the beginning and end of the algal treatment process to analyze the metabolites by mass spectrometer (MS) (1290 Infinity LC/6460 QQQ MS, Aglient). Referring to a previous study, the corresponding treatment process of 7-ACA was extended to 24 h to obtain the analyzable metabolite. In the second part, bacteriostatic assessment was carried out when the target antibiotic and its mother nucleus were treated with green algae for 6 and 24 h, respectively. The solutions of algae treated compounds at the proper times were used to cultivate *E*. *coil* and *S*. *aureus* using the spread-plate method, to observe any inhibition zones (more details in Table 1). To avoid the algal bacteriostatic effect itself, the culture solution of green algae under normal culture without antibiotic were also tested to confirm whether the algae itself provides a similar bacteriostatic effect.

The acute toxicity test for rotifers was also performed to evaluate the toxicity of ceftazidime and 7-ACA before and after the algal treatment. The toxicity assessment was performed following the guidance of ASTM, 2004. The test organism rotifer *Brachionus calyciorus* originated from a lake on the campus of China Pharmaceutical University (Nanjing, China). For the rotifer culture, the EPA medium contained 96 mg NaHCO_3_, 60 mg CaSO_4_·2H_2_O, 123 mg MgSO_4_·7H_2_O and 4 mg KCl in 1 L of deionized water at pH 7.5. In each group, 10 juveniles of the rotifer in each well were exposed to the above solutions (ceftazidime or 7-ACA). All test rotifers were incubated in darkness at 25 ± 1 °C. After a 24-h exposure without food, the number of the surviving rotifers was determined to calculate the survival rate. The rotifer cultured in EPA media served as the control. The test rotifers were considered dead if no movement of the cilia and mastax was observed after 30 s^61^. We also tested the survival rate of the rotifers exposed to the culture solution of the green algae under normal culture without any antibiotic. Each group had six replications per treatment.
